# Effect of resin infiltration system and pit-fissure sealant on white spot lesion: An *in vitro* study

**DOI:** 10.6026/973206300211169

**Published:** 2025-05-31

**Authors:** Ali Khan, Satish Maran, Rachana Jain, Shivangi Verma, Jairahadeep Satnam Singh, Madiha Khan

**Affiliations:** 1 Department of Pediatric and Preventive Dentistry, People's Dental Academy Bhopal, People's University, Bhopal, Madhya Pradesh, India; 2 Department of Pediatric and Preventive Dentistry, Daswani Dental college and Research center, Kota, Rajasthan, India; 3 Department of Pediatric and Preventive Dentistry, RKDF Dental College Bhopal, Madhya Pradesh, India; 4 Private Practitioner, New England Dental Care Massachusetts, USA

**Keywords:** ICON resin infiltration, pit and fissure sealant, white spot lesions, remineralisation

## Abstract

Demineralization causes dissolution of calcium hydroxyapatite crystals from enamel surface and leads to formation of micro-porosities resulting in chalky white patches on tooth surface thereby causing a white spot lesions. Therefore, it is of interest to compare the surface roughness, micro-hardness and masking effect of commonly used pit and fissure sealant (GC, fuji VII, IVOCLAR, helioseal F) and DMG ICON in artificial carious lesions. A total of 80 freshly extracted premolars were collected for the study. The enamel surfaces were treated using both resin infiltration and fissure sealant techniques (GC, fuji VII, IVOCLAR, helioseal F). Surface roughness was assessed with a surface profilometer, while microhardness was evaluated using the Vickers hardness test. The masking effect was determined through the Visual Analogue Scale (VAS). The highest surface roughness was observed in specimens treated with DMG ICON, followed by those treated with GC Fuji VII, while the lowest roughness was recorded by Helioseal-F in the group. In terms of microhardness, GC Fuji VII demonstrated the highest values, followed by Helioseal-F, with ICON showing the lowest. The masking ability was found to be most effective in the ICON group, which outperformed both Helioseal-F and GC Fuji VII. ICON camouflages the white spot lesions by matching the refractive index of healthy enamel. GC fuji VII helps to prevent white spot lesions by releasing fluoride, but cannot reverse or mask existing one.

## Background:

A beautiful smile, an individual's most interactive contact capacity, is developed in harmony with the gums, lips and face of the patient with pleasing intrinsic proportions to each and a pleasing teeth arrangement [1]. Throughout life, oral hygiene has an incontestable meaning. During childhood, special interventions for prevention of dental caries are enforced by general and local particularities in relation to age. Adequate nutrition, local or general fluoride use decreases the risk of dental caries in children in addition to good hygiene [2]. The most frequent chronic oral disease worldwide is dental caries. The plaque accumulation for more than two weeks would lead to the development of initial carious lesion. The Initial carious lesion may not favour the aesthetics, if they are present in the aesthetic zone they might lead to cavitation lesion in high caries risk patient if not treated appropriately [3]. White spot lesions are the initial sign of intact enamel demineralization that may or may not lead to the development of caries. The initial enamel lesions occur in the following ways, Pathogenic bacteria penetrates the enamel layer, The calcium and phosphate ions leach out by organic acids formed by the bacteria which may or may not be naturally replaced by the remineralization process [1]. This mineralized layer loss creates porosities that alter the enamel's refractive index (RI), which is usually translucent [5]. Initial carious lesion can be managed by invasive or non-invasive treatment options. The micro abrasion, composite restoration or porcelain veneers which are invasive treatment options, do not preserve the tooth structure and are indicated for aesthetic management. The conservative approach that is the non-invasive treatment option primarily focuses to remineralize the initial caries lesion. Remineralization with high concentration of topical fluoride endorses remineralization of superficial enamel layer, thus leaving the sub surface layer demineralised [6]. Over the years, several preventive agents have been studied to determine their efficacy in preventing and treating WSLs. Cements that contain fluoride (F), amorphous calcium phosphate (ACP), casein phosphopeptide or ACP [4]. Dental caries can be avoided by fluoride treatment, good oral hygiene, and healthy diet. However, these approaches are safe, but sometimes not successful, with non-compliant patients and increasing lesion progression [7].

Different methods to the removal of caries have been tried over the years, beginning with the use of a hand drill, surpassed by the treadle instrument of James Morison in 1871 [8]. Even today, traditional treatment of caries generally requires the use of a high-speed hand piece to enter the lesion and a low-speed hand piece to remove caries, i.e. primarily an invasive method. This method replaces the carious tooth structure and removes the non-carious tooth structure accidentally. A much more tissue-preserving approach to arrest and regulate the proximal or smooth carious surface lesions, namely resin infiltration, had been extensively studied in recent decades. Carious lesion resin infiltration is a micro invasive technique used for the management of initial carious lesion. The main purpose of the minimal invasive dentistry (MID) is to obscure the highly porous structures of incipient carious lesions by means of synthetic resins with low viscosity, which hinders the further bacterial and acidic attack, gives strength to the tooth structure and also improves the lesion aesthetics. So it also helps in early diagnosis by using diagnostic instruments and required minimal surgical intervention [9]. The management of resin-infiltration white spot lesions (DMG, Hamburg, Germany) is very quick, simple and painless. The patient and the doctor both support this approach well [10]. Resin infiltration seems to result in a much deeper penetration of the resin, whereas hydrochloric acid pre-treatment appears to be more acceptable compared to phosphoric acid. With regard to clinical practice, this modified etching technique is believed to reduce the effect of the highly mineralized surface layer with the potential to penetrate into lesions of fissure caries [11]. Furthermore the ability of the infiltrant to infiltrate the enamel lesions effectively may allow better clinical outcomes. Enamel caries have pores that may serve as pathways for acids and dissolved minerals to disperse. Therefore the occlusion of these pores with photo activated resins may avoid the progression of the lesion of the caries and stabilise the structurally fragile lesion mechanically [12]. A systematic analysis of *in vivo* studies showed that resin penetration appears to be a successful method of stopping the development of non-cavitated proximal caries, radiographically extended to a limit of an outer third of dentin in conjunction with non-operative steps, compared to non-operative measures applied alone [13]. For initial caries lesion resin infiltration, the resin infiltration material ICON is considered the gold standard and is highly successful in preventing caries operation and providing good aesthetic recovery [14]. ICON, however, is a very costly material and may not be affordable or protected by dental insurance for certain patients. Resin composite sealers consisting of unfilled resin are cheaper in cost as compared with ICON [15]. GC Fuji VII is high-fluoride releasing glass ionomer cement. It is commonly used as a pit and fissure sealant and preventive coating for erupting teeth [16]. Heliseal-F is a light-cured, fluoride-releasing resin. It is primarily indicated for use as a cavity liner or as a sealant in preventive dentistry [17]. The occlusal surfaces of children and adolescents at high risk for caries can be effectively protected through the prudent application of non-invasive fissure sealants, which are currently regarded as one of the most effective preventive strategies. The arrest of white spot lesions may be facilitated by the infiltration of low-viscosity; light-curing resin into subsurface enamel porosities, making resin infiltrates a promising alternative therapeutic approach [17]. To the best of our knowledge, no previous study has evaluated the surface roughness, microhardness, and masking effect of commonly used pit and fissure sealants-namely GC Fuji VII and Helioseal-F, in comparison with the gold standard resin infiltrant for carious lesion management, ICON. Therefore, it is of interest to investigate whether the surface roughness, microhardness, and esthetic masking ability of these sealants are comparable to those of ICON when applied to artificially induce initial carious lesions.

## Materials and Methods:

The freshly extracted premolars which were indicated for orthodontics purpose were obtained from the Department of Oral & Maxillofacial surgery, Peoples Dental Academy Bhopal. The freshly extracted premolars were randomly divided into 4 groups (3 experimental and 1 control group) comprising of 20 teeth in each group.

Group A: DMG ICON

Group B: Gc Fuji VII

Group C: Ivoclar Helioseal F

Group D: Control

## Materials:

[1] DMG ICON

[2] GC FUJI VII

[3] IVOCLAR HELIOSEAL F

## Data collection procedure:

[1] All the specimen teeth were stored in thymol (0.1%) solution until the day of measurement.

[2] Double sided diamond disc with profuse water irrigation were used to cut the crown longitudinally in a mesio distal direction and to remove the roots of the teeth.

[3] An (3x3mm2) area was spared and the rest surface was painted with acid resistant varnish.

[4] The entire specimens were embedded in methyl acrylate resin in such a way that it should be parallel to scanning process in all the stages.

[5] Maintaining the pH at 4.5, all the specimen were kept in demineralising solution for 4 days at room temperature [1].

[6] The demineralising solution consists of calcium chloride, potassium dihydrogen phosphate, lactic acid, sodium chloride [1].

## Surface roughness test:

Surface roughness was analyzed by using surface profilometer. The surface roughness was evaluated by using the arithmetic mean of the surface roughness profile value.

## Micro-hardness test:

The Digital Micro Vickers Hardness Tester was used to determine the microhardness of each specimen. This tester is equipped with a digital microscope; which shows measuring methods, test force, indentation length, hardness value, and dwell time of the test force as well as number of measurement, all shown on its LCD screen and evaluate the hardness values from the hardness table.

## Masking effect:

The arithmetic mean of the entire group helps to evaluate the masking effect which was used to evaluate the esthetic value of the material.

## Group A (DMG ICON):

[1] The specimen were cleaned and dried for 30 seconds.

[2] ICON DRY application was done on the lesion.

[3] ICON ETCH (15%HCL) was applied for 2 minutes on the lesion.

[4] Resin was applied on the lesion for 3 minutes and thereafter light cured.

[5] Again resin was applied for the second time & light cured for 1 minute, followed by the removal of excess material.

## Group B (Ivoclar Helioseal F):

[1] The specimen were cleaned and dried for 30 seconds.

[2] 20% phosphoric acid was used as etchant.

[3] Sealants were applied with micro brush.

[4] Sealants were self-cured after 2 minutes.

## Group C (GC Fuji Vii):

[1] The specimen were cleaned and dried for 30 second.

[2] The etchant used was 37% phosphoric acid

[3] Sealants were applied on the lesion and light cured for 2minutes.

## GROUP D (CONTROL):

The control group didn't receive any treatment, but was assessed for surface roughness, micro hardness and masking effect.

## Statistical analysis:

The data obtained was subjected to statistical analysis with the consult of a statistician. The data so obtained was compiled systematically. Statistical analysis was done using Statistical Package of Social Science (SPSS Version 22.0; Chicago Inc., USA). Data comparison was done by applying specific statistical tests to find out the statistical significance of the comparisons. Quantitative variables were compared using mean values and qualitative variables using proportions. Significance level was fixed at P < 0.05.

The following statistically test were applied,

[1] Descriptive statistics were used to calculate the mean and standard deviation of all the study group samples.

[2] ANOVA- analysis of variance were used to compare the micro-hardness, surface roughness and masking effect between the groups.

[3] Turkeys post hoc analysis was used for inter group comparison of all the study group samples.

## Results:

The numerical analysis of the micro-hardness, surface roughness and masking effect were given in the table number 1, 2, 3. The significant differences were found in surface roughness of the entire three group, the maximum surface roughness were found in the group GC fuji VII followed by DMG ICON and Ivoclar Helioseal F in decreasing order. The comparison of micro-hardness and masking effect were found statistically insignificant. Table 1 shows Comparison of various samples for Surface Roughness, a total of 80 samples were collected from this table. We concluded that maximum mean was seen in group A (2.05 ± 0.50) followed by Group C (1.59 ± 0.88) and Group D (1.09 ± 0.62) whereas lowest mean value was seen in Group B (0.88 ± 0.58). Table 2 shows Comparison of various samples of Micro hardness, from this table we concluded that maximum mean was seen in group C (254.35 ± 68.87) followed by Group B (238.35 ± 58.51) and Group D (217.30 ± 71.56) whereas lowest mean value were seen in Group A (209.10 ± 69.68). Table 3 shows Comparison of various samples of Masking Effect, from this table we concluded that maximum mean was seen in group A (7.85 ± 1.18) followed by Group B (7.25 ± 0.96) whereas lowest mean value were seen in Group C (0.55 ± 0.11). Table 4 shows Comparisons of Surface Roughness from various groups by using one way Anova test, descriptive statistics shows that that maximum mean was seen in group A (2.05 ± 0.50) followed by Group C (1.59±0.88) and Group D (1.09 ± 0.62) while lowest mean value were seen in Group B (0.88 ± 0.58) , this comparison was statistically significant. (P-value=0.002). Table 5 shows Intra group comparison by using Post Hoc analysis statistically significant results were found out in comparison of Group B with Group A, Group C, and Group D. (P-value=0.001) and comparison of Group C with another groups (P-value=0.001). Table 6 shows Comparisons of Micro hardness from various groups by using one way Anova test, descriptive statistics maximum mean was seen in group C (254.35 ± 68.87) followed by Group B (238.35 ± 58.51) and Group D (217.30 ± 71.56) whereas lowest mean value were seen in Group A (209.10 ± 69.68)., this comparison was statistically insignificant (P-value=0.145). Table 7 shows Intra group comparison by using Post Hoc analysis, from this table we concluded that all the comparisons were found to be statistically in significant (All P-value > 0.05). Table 8 shows Comparisons of Masking Effect from various groups by using one way Anova test ,descriptive statistics shows that maximum mean was seen in group A (7.85 ± 1.18) followed by Group B (7.25 ± 0.96) whereas lowest mean value were seen in Group C (0.55 ± 0.11)., this comparison was statistically significant (P-value=0.000). Table 9 shows Intra group comparison by using Post Hoc analysis statistically significant results were found out in comparison of Group A with Group B and Group C (P-value=0.000) and comparison of Group C with another groups (P-value=0.000). Figure 1 - (see PDF) shows comparison of means in various groups of Surface Roughness, maximum mean as shown in 20 samples from Group A (2.05) while lowest were seen in Group B (0.88). Figure 2 - (see PDF) shows of means in various groups for Micro hardness, maximum mean as shown in 20 samples from Group C (254.35) while lowest were seen in Group A (209.10). Figure 3 - (see PDF) shows comparison of means in various groups of Masking Effect, maximum mean as shown in 20 samples from Group A (7.85) while lowest were seen in Group A (0.55).

## Discussion:

Arresting dental caries in primary stage is very pivotal. There has been a massive evolution in caries management from surgical intervention to minimally invasive dentistry. The main objective of non-invasive and minimally invasive dentistry is dental tissue conservation by preventing dental caries and arresting its further advancement without causing destruction to the surrounding healthy tooth structure [1]. In non-invasive caries regulation is carried out using topical products such as fluoride or casein phosphor-peptide amorphous calcium phosphate which initiates the remineralization of the carious lesions. In recent years MID such as pit and fissure sealant and resin infiltration is noticeable, which required tooth surface preparation via acid and placing a sealant over the tooth surface [3]. In resin infiltration technique, pin point-sized pores within the body of carious lesion act as diffusion for acids which are infiltrated with light curing resin [1]. Thus, in this method resin with high penetration power infiltrate into the pores through capillary action and block the route of acid and fermentable carbohydrates after polymerization [2]. A promising approach for the non-operative treatment of carious lesions can be carried out by arresting the enamel lesions. The penetration of the adhesives into formerly demineralized enamel and infiltration with composite resins can lead to the arresting of the initial white spot lesions. The treatment of fissure sealant depends upon the durability of an unbroken margin between the sealant and the tooth [3].

The procedure was carried out according to the manufacturer's guidance. Teeth were cleaned with pumice and handpiece and then flushed with water spray. To achieve the high retention rates, cleaning the debris from the teeth by gently running an explorer through fissures and strenuously rinsing with water was more effective than those achieved when teeth had been cleaned by a prophy brush and pumice. The surface cleaning with toothbrush prophylaxis or a dry brushing and those with handpiece prophylaxishad same levels of sealant retention [1]. The results of the present study showed that resin infiltration (Icon) showed maximum surface roughness into initial enamel carious lesions as compared to the other groups. Arnold *et al.* stated that resin infiltration was appeared in smoothing of the acid-etched enamel surface. This smooth surface should last and further block the plaque accumulation. Triethylene glycol dimethacrylate (TEGDMA) was the Infiltrant of the resin used in this study. TEGDMA influences the water absorption and the degradation of the polymer as it has a relatively high solubility [18]. Pancu *et al.* concluded that as compared to conventional sealants Icon resin infiltrant showed greatest micro-hardness [19]. Moreover, Torres *et al.* observed that the mechanical strength of the infiltrated enamel surface was highly improved by the ability of the low-viscosity resin to fill the spaces between the remaining crystals of the porous lesion and the demineralized tissue [20]. On overall comparison of mean microhardness between the studies groups, the results of the present study showed that Group A (DMG ICON) showed minimum microhardness when compared to Group B (IVOCLAR HELIOSEAL F) and Group C (GC FUJI VII). The *in vitro* and *in vivo* studies evaluated the esthetic outcome of ICON which showed that ICON is an effective treatment option for masking initial caries lesions. In our study the mean VAS score of ICON was 7.85, followed by group B 7.25 and group C showed the least esthetic outcome, which was statistically significant. This result was consistent with Senertrao *et al.* [21]. To the best our knowledge no studies evaluated the masking ability of resin composite sealer like IVOCLAR HELOSEAL F and GC FUJI VII. From this present study we conclude that- The GC FUJI 7 can be used in partially erupted molars and in those teeth in which isolation is difficult to achieve. It shows maximum microhardness as compared to other groups but at the same time it is esthetically insignificant, so it can be used only in posterior teeth. IVOCLAR HELIO SEAL F shows minimum surface roughness, moderate microhardness and masking effect as compared to other groups, so this material can be used in anterior teeth. DMG ICON shows maximum surface roughness and moderate hardness as compared to other groups but further more research has to be done to evaluate its effect on the teeth. ICON resin infiltration penetrates subsurface porosities, altering the refractive index and masking the white spot appearance. However, the low-viscosity resin may leave a slightly rougher surface. Helioseal-F combines esthetic properties with mechanical strength, making it a balanced choice. GC Fuji VII, while clinically simple and fluoride-releasing, may be limited in cosmetic improvement due to its opacity and surface finish. In an *in vitro* study, it is difficult to achieve natural oral environment, the effect of saliva and thermocycling, further investigations are required to prevail over the limitation of the study and to check the efficacy of various materials used.

## Conclusion:

ICON resin infiltrant is effective for esthetically managing white spot lesions. It offers a micro-invasive and visually pleasing solution, especially for the anterior region. GC Fuji VII is more suitable for prevention in children and high-risk individuals due to its fluoride-releasing capability and ease of use. Ivoclar Helioseal-F, while helpful in prevention and as a liner, has limited esthetic value. An ideal clinical decision should be guided by lesion severity, location, patient age, esthetic demands, and risk factors, integrating both preventive and therapeutic strategies. Thus material selection should be guided by the clinical objective such as esthetics, fluoride release or enamel reinforcement.

## Figures and Tables

**Table 1:** Comparison of various samples for surface roughness

**Variable**	**N**	**Range**	**Minimum**	**Maximum**	**Mean**	**Std. Deviation**
Group A	20	1.5	1.02	2.52	2.05	0.5
Group B	20	1.88	0.23	2.11	0.88	0.58
Group C	20	2.72	0.46	3.18	1.59	0.88
Group D	20	1.86	0.25	2.11	1.09	0.62

**Table 2:** Comparison of various samples of Micro hardness

**Variable**	**N**	**Range**	**Minimum**	**Maximum**	**Mean**	**Std. Deviation**
Group A	20	240	116	356	209.1	69.68
Group B	20	217	116	333	238.4	58.51
Group C	20	264	116	380	254.4	68.87
Group D	20	217	116	333	217.3	71.56

**Table 3:** Comparison of various samples of masking effect

**Variable**	**N**	**Range**	**Minimum**	**Maximum**	**Mean**	**Std. Deviation**
Group A	20	4	6	10	7.85	1.18
Group B	20	3	6	9	7.25	0.96
Group C	20	1	0	1	0.55	0.11

**Table 4:** Comparisons of surface roughness from various groups by using one way anova test

**Variable**	**N**	**Mean**	**Std. Deviation**	**95% Confidence Interval for Mean**		**F**	**P-value**
				**Lower Bound**	**Upper Bound**		
Group A	20	2.05	0.5	1.81	2.293	12.534	0.002
Group B	20	0.88	0.58	0.61	1.1571		
Group C	20	1.59	0.88	1.18	2.0122		
Group D	20	1.09	0.62	0.8	1.3852		
Total	80	1.4	0.79	1.23	1.5854		

**Table 5:** Intra group comparison of surface roughness by using Post Hoc analysis

**Variable**		**Mean Difference**	**95% Confidence Interval**		**Sig.**
			**Lower Bound**	**Upper Bound**	
Group A	Group B	1.17	0.7537	1.5909	0.033
	Group C	0.45	0.0392	0.8764	
	Group D	0.96	0.5449	1.3821	
Group B	Group A	-1.17	-1.59	-0.75	0.001
	Group C	-0.71	-1.13	-0.29	
	Group D	-0.2	-0.62	0.2	
Group C	Group A	-0.45	-0.8764	-0.0392	0.001
	Group B	0.71	0.296	1.1331	
	Group D	0.5	0.0871	0.9243	
Group D	Group A	-0.96	-1.38	-0.54	0.324
	Group B	0.2	-0.2	0.62	
	Group C	-0.5	-0.92	-0.08	

**Table 6:** Comparisons of micro hardness from various groups by using one way anova test

**Variable**	**N**	**Mean**	**Std. Deviation**	**95% Confidence Interval for Mean**		**F**	**P-value**
				**Lower Bound**	**Upper Bound**		
Group A	20	209.1	69.68493	176.4864	241.7136	18.5	0.145
Group B	20	238.4	58.5116	210.9657	265.7343		
Group C	20	254.4	68.8708	222.1175	286.5825		
Group D	20	217.3	71.56013	183.8088	250.7912		
Total	80	229.8	68.43087	214.5465	245.0035		

**Table 7:** Intra group comparison of micro hardness by using Post Hoc analysis

**Variable**		**Mean Difference**	**95% Confidence Interval**		**Sig.**
			**Lower Bound**	**Upper Bound**	
Group A	Group B	29.25	71.66	13.16	0.037
	Group C	45.25	87.66	28.32	
	Group D	82.08	50.61	34.21	
Group B	Group A	29.25	13.16	71.66	0.455
	Group C	16	58.41	26.41	
	Group D	21.05	21.36	63.46	
Group C	Group A	45.25	28.32	87.66	0.086
	Group B	16.09	26.41	58.41	
	Group D	37.05	53.69	79.46	
Group D	Group A	28	-34.21	50.61	0.326
	Group B	21.05	-63.46	21.36	
	Group C	37.05	-79.46	36.8	

**Table 8:** Comparisons of masking effect from various groups by using one way Anova test

**Variable**	**N**	**Mean**	**Std. Deviation**	**95% Confidence Interval for Mean**		**F**	**P-value**
				**Lower Bound**	**Upper Bound**		
Group A	20	7.85	1.18	7.29	8.4	6.16	0
Group B	20	7.25	0.96	6.79	7.7 0		
Group C	20	0.55	0.51	0.31	0.78		
Total	60	5.21	3.45	4.32	6.11		

**Table 9:** Intra group comparison of masking effect by using Post Hoc analysis

**Variable**		**Mean Difference**	**95% Confidence Interval**		**Sig.**
			**Lower Bound**	**Upper Bound**	
Group A	Group B	0.6	0.011	1.18	0
	Group C	7.3	6.711	7.88	
Group B	Group A	0.68	13.16	71.66	0.04
	Group C	6.7	58.41	26.41	
Group C	Group A	7.3	7.88	6.71	0
	Group B	6.7	7.28	6.11	
